# Supplementary material for: Evidence supported by Mendelian randomization: impact on inflammatory factors in knee osteoarthritis
Source: Front Med (Lausanne). 2024 May 28;11:1382836. doi: 10.3389/fmed.2024.1382836 (PMC11165061; doi:10.3389/fmed.2024.1382836)
Supplement: Supplementary file 1 [file Data_Sheet_1.ZIP › data/GCST90274776_5e6/again/776.leaveoneout.pdf]

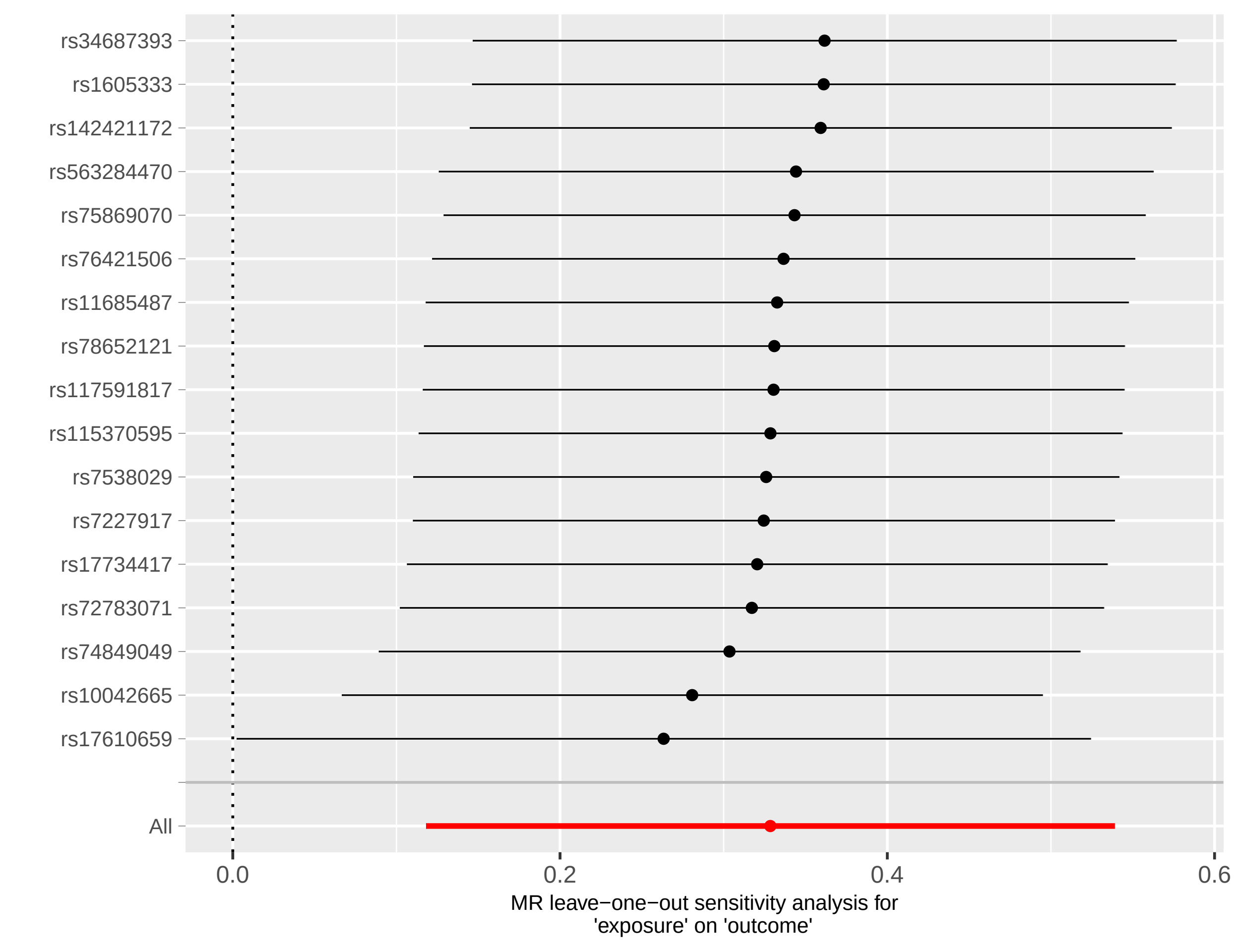
rs34687393
rs1605333
rs142421172
rs563284470
rs75869070
rs76421506
rs11685487
rs78652121
rs117591817
rs115370595
rs7538029
rs7227917
rs17734417
rs72783071
rs74849049
rs10042665
rs17610659
All
0.0
0.2
0.4
0.6
MR leave−one−out sensitivity analysis for
'exposure' on 'outcome'
